# D-PLACE: A Global Database of Cultural, Linguistic and Environmental Diversity

**DOI:** 10.1371/journal.pone.0158391

**Published:** 2016-07-08

**Authors:** Kathryn R. Kirby, Russell D. Gray, Simon J. Greenhill, Fiona M. Jordan, Stephanie Gomes-Ng, Hans-Jörg Bibiko, Damián E. Blasi, Carlos A. Botero, Claire Bowern, Carol R. Ember, Dan Leehr, Bobbi S. Low, Joe McCarter, William Divale, Michael C. Gavin

**Affiliations:** 1 Department of Ecology & Evolutionary Biology, University of Toronto, Toronto, Canada; 2 Department of Geography & Planning, University of Toronto, Toronto, Canada; 3 Max Planck Institute for the Science of Human History, Jena, Germany; 4 School of Psychology, University of Auckland, Auckland, New Zealand; 5 ARC Centre of Excellence for the Dynamics of Language, ANU College of Asia and the Pacific, Australian National University, Canberra, Australia; 6 Department of Archaeology and Anthropology, University of Bristol, Bristol, United Kingdom; 7 Department of Comparative Linguistics, University of Zürich, Zürich, Switzerland; 8 Psycholinguistics Laboratory, University of Zürich, Zürich, Switzerland; 9 Department of Biology, Washington University, Saint Louis, MO, United States of America; 10 Department of Linguistics, Yale University, New Haven, CT, United States of America; 11 Human Relations Area Files, Yale University, New Haven, CT, United States of America; 12 Center for Genomic and Computational Biology, Duke University, Durham, United States of America; 13 University of Michigan School of Natural Resources & Environment, Ann Arbor, MI, United States of America; 14 University of Michigan Institute for Social Research, Ann Arbor, MI, United States of America; 15 Center for Biodiversity and Conservation, American Museum of Natural History, New York, NY 10024, United States of America; 16 York College, City University of New York, New York, United States of America; 17 Department of Human Dimensions of Natural Resources, Colorado State University, Fort Collins, CO, United States of America; University of Exeter, UNITED KINGDOM

## Abstract

From the foods we eat and the houses we construct, to our religious practices and political organization, to who we can marry and the types of games we teach our children, the diversity of cultural practices in the world is astounding. Yet, our ability to visualize and understand this diversity is limited by the ways it has been documented and shared: on a culture-by-culture basis, in locally-told stories or difficult-to-access repositories. In this paper we introduce D-PLACE, the Database of Places, Language, Culture, and Environment. This expandable and open-access database (accessible at https://d-place.org) brings together a dispersed corpus of information on the geography, language, culture, and environment of over 1400 human societies. We aim to enable researchers to investigate the extent to which patterns in cultural diversity are shaped by different forces, including shared history, demographics, migration/diffusion, cultural innovations, and environmental and ecological conditions. We detail how D-PLACE helps to overcome four common barriers to understanding these forces: i) location of relevant cultural data, (ii) linking data from distinct sources using diverse ethnonyms, (iii) variable time and place foci for data, and (iv) spatial and historical dependencies among cultural groups that present challenges for analysis. D-PLACE facilitates the visualisation of relationships among cultural groups and between people and their environments, with results downloadable as tables, on a map, or on a linguistic tree. We also describe how D-PLACE can be used for exploratory, predictive, and evolutionary analyses of cultural diversity by a range of users, from members of the worldwide public interested in contrasting their own cultural practices with those of other societies, to researchers using large-scale computational phylogenetic analyses to study cultural evolution. In summary, we hope that D-PLACE will enable new lines of investigation into the major drivers of cultural change and global patterns of cultural diversity.

## Introduction

Human cultural diversity is expressed in myriad ways. Collectively we speak thousands of different languages, engage in hundreds of different religious practices, and abide by a diverse array of marital, sexual, and child-rearing norms. Humans have diverse ways of categorizing the natural and social world, from names of colours to body parts to whom we call kin. We build different types of houses, exploit different resources for subsistence, and we have multiple means of resource management, political institutions and economic organization. These cultural features vary across space and time, but the factors and processes that drive cultural change and shape the patterns of diversity remain largely unknown.

Dating back to at least the 19^th^ century, multiple academic disciplines have debated the degree to which patterns in cultural diversity are shaped by different forces, including shared history, demographics, genetics, migration, human inventions, environmental and ecological conditions [1–3]. However, four barriers have prevented advances in our understanding of cultural diversity patterns. The first lies in the location and dispersal of the data, which are spread across multiple disparate sources in the fields of anthropology, linguistics, ecology, and biogeography. The second barrier is the fact that these datasets use an array of ethnonyms to describe cultural groups and languages, preventing straightforward linkages between cultural and linguistic information. Third, different databases have different definitions of cultural groups and distinct time and place foci for their coded information. Finally, without ready access to relevant environmental, geographic and linguistic data, researchers cannot easily explore spatial or phylogenetic dependencies among cultural groups (Tobler’s First Law; Galton’s problem). As a result, analyses may be less likely to account for the non-independence of cultural groups or consider uncertainty in historical dependencies among groups [4,5].

**Fig 1:**
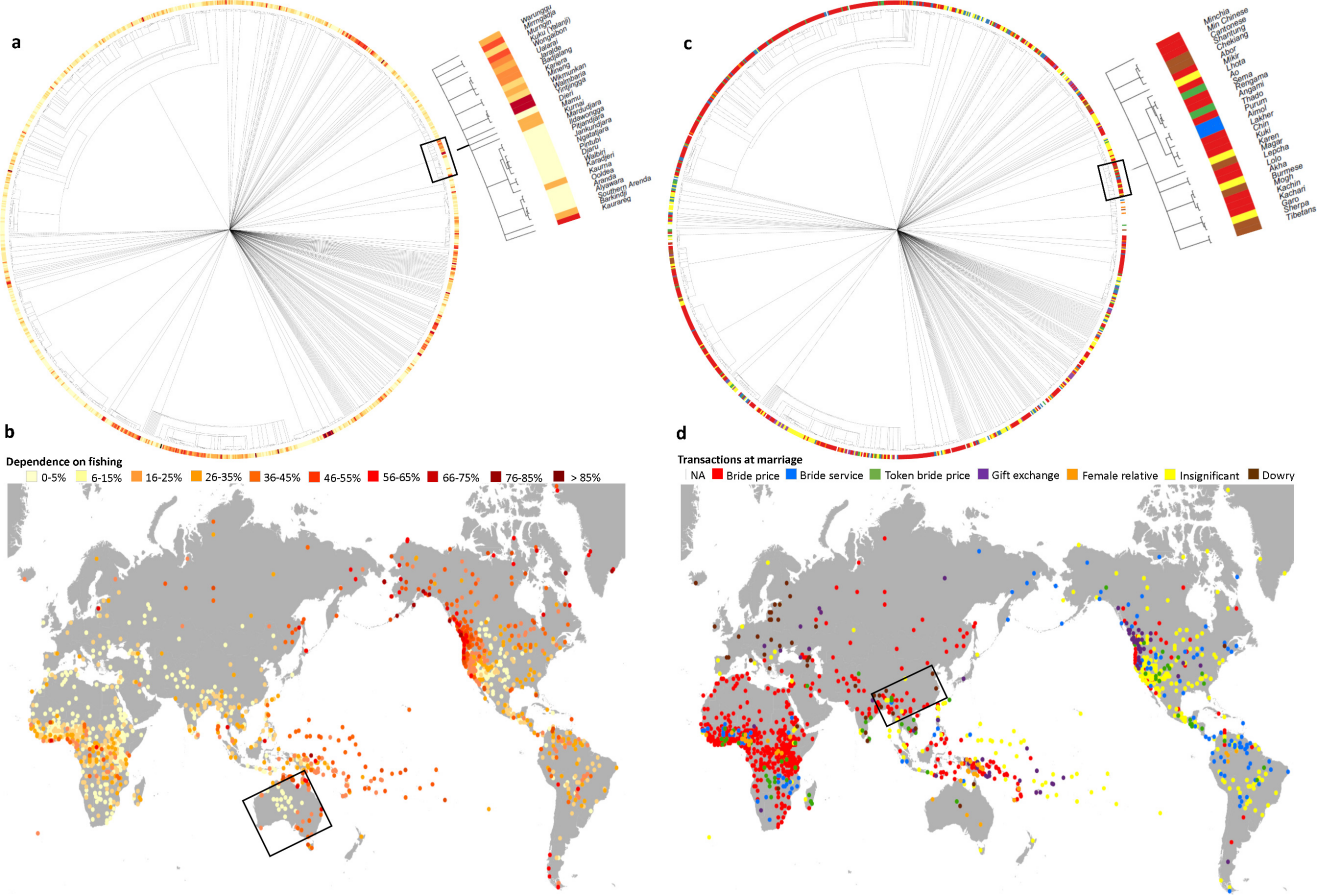
**D-PLACE links cultural information to language classifications and phylogenies (a, c) and to geographic locations and environmental features (b, d). This allows users to consider the relative influence of cultural ancestry, spatial proximity, and environment on diverse cultural practices. For example, panels a and b illustrate variation among societies in their dependence on fishing relative to other subsistence activities, based on data from the Ethnographic Atlas (EA)** [11–15] **and the Binford Hunter-Gatherer dataset** [16,17]. **Panels c and d highlight diversity in the most common economic transaction at marriage, based on data from the EA. In addition to providing global results, D-PLACE allows users to focus a search on a particular geographic region or linguistic family. Here, results for societies speaking Pama-Nyungan languages (a, b) or Sino-Tibetan languages (c, d) are magnified and outlined in black boxes on the global tree and map.**

Here we introduce D-PLACE: the Database of Places, Language, Culture, and Environment (https://d-place.org). D-PLACE solves all four challenges above by connecting a wide variety of cultural information for over 1400 human “societies” or ethnolinguistic groups with language classifications and phylogenies, and with data on geographical location and environmental features. In allowing users to map cultural features of ethnolinguistic groups onto language phylogenies, the cultural information in D-PLACE can be used in conjunction with comparative methods recently adapted from evolutionary biology [6] to examine cultural change while accounting for Galton’s problem (Fig 1A and 1C) [7,8]. Cultural features can also be mapped in space with D-PLACE, and linked to various environmental variables at those locations (e.g., [9,10]; Fig 1B and 1D). Combining language phylogenies with data on cultural features and environmental variables permits a whole new line of investigation into the possible drivers of cultural change and resulting patterns of cultural diversity. D-PLACE is also expandable, allowing new sets of data to be added to increase the scope and reach of the database.

### New frontiers in cross-cultural research

**Fig 2:**
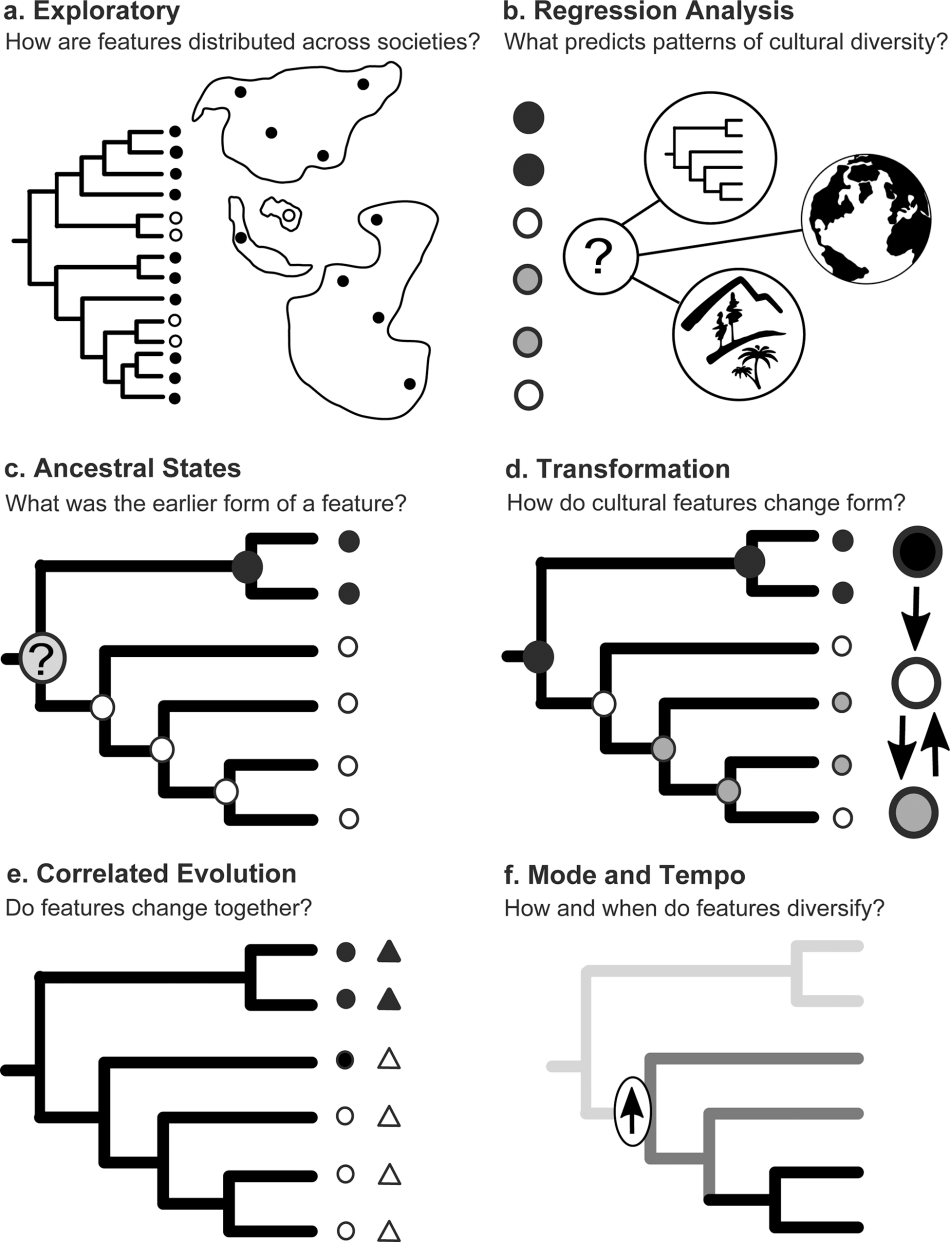
An overview of some of the types of studies that the Database of Places, Language, Culture, and Environment (D-PLACE) may facilitate.

D-PLACE facilitates three main categories of cross-cultural studies: exploratory, predictive, and evolutionary analyses (Fig 2). Here we briefly outline these different categories of study and provide examples of the data, tools, and outputs associated with each (Table 1).

**Table 1:** Examples of data and tools relevant to the different types of analyses made possible by D-PLACE.

Type of Analysis	Relevant Data	Possible tools for analysis	Example
**Exploratory** (Figs 1 and 2A)	Cultural features, environment, taxonomy or phylogeny, spatial location	D-PLACE search by language group, geographic region, and/or environmental or cultural variable, with results displayed on DPLACE map or tree	See Fig 1
**Prediction** (i.e., Regression analysis; Fig 2B)	Cultural features, environment, taxonomy or phylogeny, spatial location	lme4 [22], APE [23], MCMCglmm [24],regression packages included with other statistical software	Refs [10,25]
**Ancestral state reconstruction** (Fig 2C)	Phylogeny, cultural features	APE [23], Phytools [26], Bayestraits [27], Diversitree [28]	Ref [29]
**Transformation models** (Fig 2D)	Phylogeny, cultural features	APE [23], Phytools [26], Bayestraits [27], Diversitree [28]	Refs [19,30]
**Correlated evolution** (Fig 2E)	Phylogeny, cultural features, environment	APE [23], Phytools [26], Bayestraits [27]	Refs [20,31]
**Modes/tempo** (Fig 2F)	Phylogeny, cultural features	APE [23], Phytools [26], Diversitree [28], Bamtools [32]	Ref [21]

#### Exploration

Which cultural groups are polygynous? Where do people build circular homes? What are the subsistence strategies used by the Hadza? We anticipate users of D-PLACE to extend beyond scholars, and to include high-school and university students as well as members of the public in diverse communities around the world. Users can both download data and use D-PLACE’s display tools online to explore how cultural features and environmental variables are distributed across cultural groups and geographic regions (Figs 1 and 2A).

#### Prediction

Using data in D-PLACE, researchers can also examine associations among variables and possible predictors of variations in cultural features (Fig 2B). For example: Does polygyny correlate with women’s contribution to labour [18]? Are raised homes more likely to exist in areas of high rainfall? To what degree does history (i.e. shared ancestry), geography or environment predict different cultural features?

#### Evolution

Evolutionary approaches require an understanding of historical relationships among cultural groups. D-PLACE allows users to map cultural features onto either language classifications (i.e. trees that depict the historical relationships among languages) or phylogenies (i.e. language trees that also indicate the distances between languages). These data can then be used to undertake four different types of evolutionary analysis (Fig 2C–2F; Table 1): ancestral state reconstruction (e.g., what was the ancestral pattern of post-marital residence for Austronesian people?), cultural transformation analysis (e.g., does communal land tenure always precede private land tenure? [19]; correlated evolution (e.g., do defendable resources and inheritance systems co-evolve? [20]; and analysis of the tempo of evolution (e.g., are some features linked to increases in the rates of cultural change and diversification? [21].

### A new era of cross-cultural analysis

D-PLACE brings together a wealth of cultural data that had previously only been available in disparate and relatively inaccessible repositories. In linking these data to particular ethnographic sources and to a focal time and place, D-PLACE enables users to define their own cultural “units” and to decide how these units and their associated data should be combined for cross-cultural analysis. Linking the wealth of information on human cultural diversity to language classifications, geographic location and environmental features enables hypotheses to be rigorously tested using a rapidly growing suite of statistical and computational tools. We have made D-PLACE available as an extensible resource, and invite appropriate contributions of new phylogenies and newly-coded cultural data. We hope that D-PLACE will enable a whole new generation of scholars to answer long-standing questions about the forces that have shaped human cultural diversity.

## Database Content

### Cultural data

To date, D-PLACE includes coded cultural data drawn from two major cross-cultural databases: the Ethnographic Atlas [11–15] and the Binford Hunter-Gatherer dataset [16,17]. The Ethnographic Atlas was chosen as a starting point because, with 1291 societies, it is the largest of the different cross-cultural databases (see a comparison of samples in [33,34]). As cultural features are dynamic and often display internal variation, most cross-cultural researchers have coded variables for a particular time and place focus [33,34]. D-PLACE facilitates the matching of time and place foci among datasets that are compiled by different authors by ensuring that downloaded data are tagged with a focal time (the year to which ethnographic data refer), and focal place that includes a focal latitude/longitude and any supplementary information provided on location (e.g., name of village or area). In addition, each data point is linked to one or more of the 4,000+ ethnographic sources that were consulted in coding the data [11–14,16]. In preparing the EA and Binford datasets for D-PLACE, we replaced society names identified as pejorative with a preferred, English-language ethnonym. A searchable list of ‘alternate’ names for each society includes the original society name and, where available, one or more autonyms in the society’s own language, as well as other commonly encountered ethnonyms.

For heuristic purposes, we use the term “society” to refer to cultural groups in the database. In most cases, a society can be understood to represent a group of people at a focal location with a shared language that differs from that of their neighbors. However, in some cases multiple societies share a language (S1 Table). There is also some variation among authors of different datasets in how societies are delineated, with the same cultural group embedded in a larger unit in one cross-cultural sample, but split into multiple groups in another. For example, the society Murdock [11] refers to as “Tunava” includes both the Deep Springs Valley and Fish Lake Valley Paiute groups, whereas Binford [16] describes the Fish Lake and Deep Springs Paiute as distinct societies. As described below, D-PLACE highlights potential links among such societies by assigning them a matched “cross-dataset id”, but leaves decisions on how to combine data to the user.

Here we briefly describe the two component databases. S1 Supporting Information provides additional details on the methods we used to adapt the Ethnographic Atlas and Binford Hunter-Gatherer dataset for inclusion in D-PLACE.

#### Ethnographic Atlas database

D-PLACE includes coded data from the Ethnographic Atlas (EA) for 1291 societies distributed globally (Fig 1), ranging from societies with complex agricultural economies and political systems to small hunter-gatherer groups [11–15]. The EA focuses on preindustrial societies, not on contemporary nation-states. Over 90 cultural traits are coded in the EA, with an emphasis on those describing kinship and marriage, but including traits describing subsistence economy, religion, and the division of labour. The “focal year”, i.e., the time period to which the cultural data refer is before 1800 for 3% of societies, in the 19th century for 25%, between 1900 and 1950 for 69%, and after 1950 for 2%; 1% of the 1291 societies are missing a focal year. While the sample is global, there is an emphasis on North American and African societies.

#### Binford Hunter-Gatherer database

The Binford Hunter-Gatherer database includes coded cultural data for 339 hunter-gatherer societies [16]. According to Binford ([16]:130), the sample includes “all hunter gatherer groups known to exist during colonial and more recent era […] that were described with sufficient detail to be included in a comparative analysis.” The database includes 40 ethnographic variables, some of which overlap topically with those of the EA (e.g., subsistence economy, marriage system), and others that are distinct (e.g., size of groups cooperating for subsistence, distance moved by nomadic societies per year). The focal year for data in the Binford dataset is before 1800 for 2% of societies, in the 19th century for 63%, between 1900 and 1950 for 22%, and after 1950 for 11%; 2% of the 339 societies are missing a focal year [17]. Of the Binford societies 66% are also described in the EA, though in some cases their focal dates and locations differ from their EA counterparts. Compared to the EA, the Binford dataset includes many more societies in Australia and in northern North America.

#### Combining cultural data across the EA and Binford datasets

We have not attempted to combine cultural data across different contributing databases, for two reasons. First, even when working on similar topics, different ethnographers may have had particular emphases, and different coders/authors may have unique coding scales and rules. Second, as noted above, different authors have often used different time and place foci even though they are coding the same society. Because cultural practices change over time and vary by region, discrepancies are to be expected when the foci are different. For example, both the EA and Binford datasets include cultural data for the Pumé (“Yaruro”) of Venezuela. Recent ethnographies distinguish between River Pumé and Savanna Pumé, with River Pumé described as more dependent on horticulture, and Savanna Pumé on foraging [35]. The EA and Binford datasets differ in their foci for the Pumé, and the values Murdock and Binford assigned to hunting, gathering and fishing as sources of Pumé subsistence diverge accordingly. The EA, which relies on descriptions of Pumé of the Cinaruco River by Leeds [36], describes Pumé subsistence as made up of a near-equal mix of shifting agriculture combined with pig husbandry (contributing approximately 40% and 10% to subsistence, respectively) and hunting-gathering-fishing (contributing 20%, 20% and 10%, respectively). The Binford dataset describes hunting, gathering and fishing as contributing 6%, 41%, and 53% of subsistence needs, respectively, reflecting Binford’s greater reliance on work carried out with Savanna Pumé (e.g., [37]). Many similar examples exist, and therefore we have chosen to present data from the EA and Binford datasets separately in D-PLACE and allow users to decide how best to combine these different data sources for their intended purposes.

Differences in time foci can also be critical. For example, the main focal year for matched Binford and EA societies sometimes differs by more than 50 years (e.g., the focal year for Chumash in the EA is 1800, and in Binford is 1860). Users may therefore wish to consider whether discrepancies in codes could reflect cultural changes between the focal times described. The Binford dataset is one of the few major cross-cultural datasets to report multiple estimates for different time and place foci for a single society. For example, Binford ([16]:288–298) provides estimates of household size pre- and post-settlement in reservations for some societies in the US Southwest; in summer vs. winter for arctic groups; in the wet vs. dry season for tropical groups; and in different settlements or villages of the same society. In deciding not to harmonize or summarize these data in any way, D-PLACE maintains the insights into intra-cultural variation they provide. For display on the website’s maps and trees, one estimate is chosen at random for each society. All estimates are included when data are downloaded as a comma-separated values (CSV) file.

We provide users with a number of tools to help make decisions about when and where cultural data may be compared and combined. First, as mentioned previously, data are tagged with a society name, the dialect or language spoken by the society, a focal time, and a focal place. Second, each cultural data point is linked to its source ethnographies where possible. Third, to facilitate access to further cultural data for D-PLACE societies, we also provide information on where each society appears in other major cross-cultural databases, including the Standard Cross-Cultural Sample (see [38]; see [39]); eHRAF World Cultures (HRAF; [40]); Jorgensen’s Western North American Indian dataset [41], and Bowern’s CHIRILA dataset for Australian languages [42].

While differences in time and place foci are undoubtedly important sources of variation in the data, biases of dataset coders and of the ethnographers on whose descriptions codes are based will also be important. We therefore urge researchers thinking of using variables in D-PLACE for new research to consult the detailed codebooks that are linked to each component database, as these provide complete descriptions of coding rules used by Murdock and Binford, as well as any decisions made by D-PLACE authors when adapting the codes for D-PLACE (see also S1 Supporting Information). We also recommend researchers consider coding a random sample of the societies from the original ethnographic sources to assess inter-coder reliability, and to better understand the source ethnographies on which the codes are based.

### Linguistic Data

The language spoken by a society is an important indicator of historical relatedness, cultural identity and contact. D-PLACE specifies the broad language family affiliation for all societies, using the classification systems of Glottolog (glottolog.org; [43]). Users can treat language family as a variable of interest itself, or can use it as a coarse-level control for relatedness among societies (e.g., [10]). S1 Table summarizes the number of societies per language family in D-PLACE so far.

At a closer resolution, all societies in D-PLACE have been linked to a language and, in cases where the language was shared with another D-PLACE society, to a Glottolog dialect. Languages are identified by both a Glottolog ID and an ISO 639–3 code, and dialects by a Glottolog ID [43,44]. For languages for which an ISO 639–3 code has not been assigned, we use a D-PLACE serial number as a place-holder (x01, x02…; all within the ISO-639-3 private use range). Languages and dialects are used by D-PLACE to link each society to Glottolog’s language classification trees. These trees are topological only, representing genealogical hypotheses of how languages are nested, based on comparative historical linguistic work. The classifications are purely taxonomies and branch lengths do not represent time or amount of change.

At the finest scale, many of the societies in each database belong to a language family for which a well-resolved and computationally-derived phylogenetic tree is available (for example: [21,45–53]). In focusing analyses on these societies, researchers gain the ability to conduct sophisticated hypothesis testing about evolutionary change using phylogenetic comparative methods, as well as robust control for historical relatedness. For example, the relative time since language divergence can be used as a measure of relative distance among societies. Of course, while language provides a highly effective proxy for shared history, language family affiliation may not always reflect deep cultural or linguistic ancestry. Numerous instances of language shift, contact, and borrowing occur when societies interact. For example, many Central African Pygmy groups have adopted the languages of their Bantu trading partners [54]. In such cases, linguistic relationships still capture meaningful aspects of cultural interaction, but users will need to make their own context-specific judgments.

We triangulated language-to-society matches using a combination of bibliographic information from the original EA and Binford databases, digital sources (especially Ethnologue.com [55], MultiTree.org [56], and glottolog.org [43], geographic information (coordinates for each society were compared to coordinates for languages in the World Language Mapping System [57] and Glottolog), and input from linguists (C. Bowern, Pers. Comm., M. Dunn, Pers. Comm., H. Hammarström, Pers. Comm., H. Haynie, Pers. Comm.). Multilingual societies were linked to their most commonly spoken language. When a computationally-inferred phylogeny for a language family was available, we used society-language matches to map societies to the “tips” of the phylogenetic trees. A few of these phylogenies are well-represented by societies in the EA and Binford databases (such as Austronesian and Bantu; S1 Table) highlighting the potential for D-PLACE to be used in analyses of multiple cultural features and their inter-relationships (e.g. [31,58]).

### Environmental Data

We sampled environmental variables at the localities reported in the Ethnographic Atlas and Binford Hunter-Gatherer dataset, with some adjustments to geographic coordinates as outlined in S1 Supporting Information. Because a vast majority of societies were sampled between 1901 and 1950, we attempted to sample environmental variables at each locality for this particular time period. For each society, we computed mean, variance, and predictability of annual cycles of precipitation, temperature, and net primary productivity; number of species of birds, mammals, amphibians, and vascular plants, as well as ecoregion, biome, elevation and slope of the location (see S1 Supporting Information for sources). Contemporary values are reported for variables in cases for which the optimal range of historical data was not available. Any deviations from the target time period or from a society’s reported location are recorded in a comment field.

## Database Structure

The cultural, environmental, linguistic and geographical data in D-PLACE are stored in the open-source relational database PostgreSQL as a series of normalised tables linked by foreign keys. In order to store language and culture names correctly, all information is encoded in the Unicode format UTF-8. D-PLACE is implemented in the programming language *Python* and the open-source web-development framework *Django* (http://www.djangoproject.com). Geographical functionality is provided by the PostGIS library for PostgreSQL. The relational structure and component tables of the database are illustrated in S1 Fig and briefly described below.

The ***Society*** table stores basic information on societies. Each society has a unique identifier, a name, a list of alternative names, a main focal year, a link to its dataset source, a location (latitude/longitude stored as a PointField coordinate), an ‘original location’ field (latitude and longitude given for the society in the original source, without corrections described in S1 Supporting Information), and a link to a geographic region. Each society also has a “cross-dataset” identifier (xd_id), which is used to link societies present in different datasets.

The ***GeographicRegion*** table contains information on geographic regions. Each geographic region contains a unique numeric ID, a region name, the continent name, a *Biodiversity Information Standards* (TDWG) code, and a geometric field.

All sources are stored in the ***Source*** table, which labels each source with a unique identifier and includes fields for year, author, and the full reference for the source.

Environmental and cultural data are stored separately. At the highest level, records and variables are grouped by thematic category, with categories designed to help users narrow their searches to variables of interest (e.g., users can search for all variables relating to “Climate”, or “Kinship”). Records are then linked to a specific variable (e.g., “Mean annual temperature,” “Economic transactions at marriage”), and finally to a value and/or code (e.g., “15°C,” “Bride wealth”). In the case of cultural data, codes are further linked to individual code descriptions.

The ***EnvironmentalCategory*** table stores environmental categories, while information about individual environmental variables is stored in the ***EnvironmentalVariable*** table. This includes the variable name, units, and a description of the variable. Each environmental variable in the *EnvironmentalVariable* table is linked to a category in the *EnvironmentalCategory* table.

The ***Environmental*** table links environmental data to societies. Each row in the *Environmental* table is linked to a society in the *Society* table. Each environmental record also has a comment field, in which we have documented any adjustments made to either the target location (i.e., to a society’s lat/long) when extracting environmental data, or to the target time period of 1900–1950 when extracting climate data.

The ***EnvironmentalValue*** table stores the environmental values in D-PLACE. Each value is linked to a record (and thus to a society) in the *Environmental* table, to an environmental variable in the *EnvironmentalVariable* table, and also has a coded value.

Cultural categories are stored in the ***CulturalCategory*** table, and cultural variables in the ***CulturalVariable*** table. Each cultural variable description is linked to its dataset source in the *Source* table, and has a label (e.g., EA070), a name, a description, a data type (Continuous, Ordinal). Variable descriptions are linked to variable categories (many-to-many) in the *CulturalCategory* table.

The discrete values used to code variables in the datasets are stored in the ***CulturalCodeDescription*** table. This table contains the complete definition of each cultural code (e.g., “Polygynous, with polygyny occasional or limited”), a shortened code description for display in map and tree legends (e.g., “Limited polygyny”), and the code number (e.g., “2”). Each variable code is also linked to a variable in the *CulturalVariable* table.

All coded cultural data is stored in the ***CulturalValue*** table. Each coded value is linked to a variable in the *CulturalVariable* table, a society in the *Society* table, and a code in the *CulturalCodeDescription* table. Each data point stored in the *CulturalValue* table is also linked to references in the *Source* table via a many-to-many field. Each coded value also has a comment, a field for supplementary information on location (e.g., village name), and a field for specific year, to allow for deviations from the ‘main’ focal year for the society.

Language information is stored in the ***Language*** table. Each society is linked via its ‘cross-dataset identifier’ (xd_id) to an ISO-639-3 language code, a Glottolog language or dialect ID, and a Glottolog dialect or language name. The ***LanguageFamily*** table contains information on each language’s largest genealogical unit—usually a linguistic family or the language itself when there are no attested related languages. The table includes a field for the name of this unit and a field indicating the classification scheme used to assign languages to units. Currently, the only scheme used in D-PLACE to assign languages to language families (and to identify language isolates) is that of Glottolog. All language trees are stored, in Newick format, in the ***LanguageTree*** table. Each language tree has a name, the Newick string, and is linked (via a many-to-many field) to languages in the *Language* table.

In summary, the database is structured to facilitate future additions of coded cultural data, and to allow linguistic and environmental data to be updated as new phylogenies and datasets become available. The Max Planck Institute for the Science of Human History has committed to the long-term hosting and maintenance of D-PLACE, ensuring it will remain accessible to cross-cultural researchers.

## Data Visualization

The user interface allows users to search for societies via geographic region, cultural trait, environmental variable, or language. D-PLACE has been designed to be accessible to different user communities with a straightforward user interface. In addition to being summarized in a table, search results can be displayed on a map, language phylogeny or Glottolog tree. Advanced users may also download datasets for offline analysis.

### Map view

Two maps use the Biodiversity Information Standards Geographic Regions Level 2 shapefile, which divides the world into major regions (e.g., Australia, Northern Africa, Siberia) [59]. The shapefile was converted to javascript using jVectorMap. Societies were then linked to the map using their geographic coordinates, and users can search for societies by region by clicking on the appropriate section of the map.

Maps also allow users to visualize search results for environmental, cultural and language family data in space. Markers for each society are displayed on a zoomable map and coloured according to their coded value. Only one variable can be displayed on the map at once. Maps can be downloaded as svg images.

### Phylogeny view

Language trees are available in two formats–Glottolog trees and Bayesian phylogenic trees. Glottolog trees are taxonomies, rather than time-calibrated phylogenies. While this limits analyses because branch lengths are not calibrated to time, they are available for most of the world’s language families. In contrast, time-calibrated Bayesian phylogenetic trees are currently only available for societies speaking Austronesian, Bantu, Dene-Yeniseian, Indo-European, Japonic, Koreanic, Pama-Nyungan, Semitic, and Tukanoan languages. We therefore provide users with the option of mapping features onto Glottolog taxonomies (all societies) or Bayesian phylogenies for select families. In the future we expect to increase the number of computationally-inferred phylogenies in D-PLACE as more become available in the literature.

The near-global coverage of Glottolog allows users to view results on a ‘global tree’. The global tree links all component Glottolog family trees to a common ancestor without making any assumptions about relationships among component families. The global tree allows users to zoom in/out of individual sections (e.g., Fig 1A and 1C). Glottolog trees were downloaded from glottolog.org in Newick format. Phylogenies were made available by their respective authors for inclusion.

All trees are displayed on the website using d3js, a javascript library used to visualize data. Trees are stored in the database in Newick format, and were parsed for display using Newick.js. Languages not spoken by societies in D-PLACE were pruned using Python’s ete2 library. Coded values were linked to tree tips for display using language codes. In cases where more than one society shares a language, one society is chosen at random for display. As with the maps, trees can be downloaded as Scalable Vector Graphic (SVG) images.

## How to Cite D-PLACE

Research that uses data from D-PLACE should cite both the original source(s) of the data and this paper (e.g., research using cultural data from the Binford Hunter-Gatherer dataset: “Binford (2001); Binford and Johnson (2006); Kirby et al. 2016).” The reference list should include the date that data were accessed and URL for D-PLACE (http://d-place.org), in addition to the full reference for Binford (2001) and Binford and Johnson (2006).

## Supporting Information

S1 Fig Database structure. (PDF)

S1 Supporting information D-PLACE data and sources. (PDF)

S1 Table D-PLACE societies per language family. Currently, D-PLACE contains cultural data for over 1400 societies, drawn from two major cross-cultural datasets (the Ethnographic Atlas and Binford Hunter-Gatherer datasets). The societies are associated with 1202 unique languages and approximately 1315 dialects. Linguistic information for each society is available for download through D-PLACE, with all languages and dialects linked to Glottolog identifiers (glottolog.org; [43]).
(PDF)
