# Supplementary material for: D-PLACE: A Global Database of Cultural, Linguistic and Environmental Diversity
Source: PLoS One. 2016 Jul 8;11(7):e0158391. doi: 10.1371/journal.pone.0158391 (PMC4938595; doi:10.1371/journal.pone.0158391)

S2 Figure. Database structure. Boxes in the schema below represent tables in the relational database.

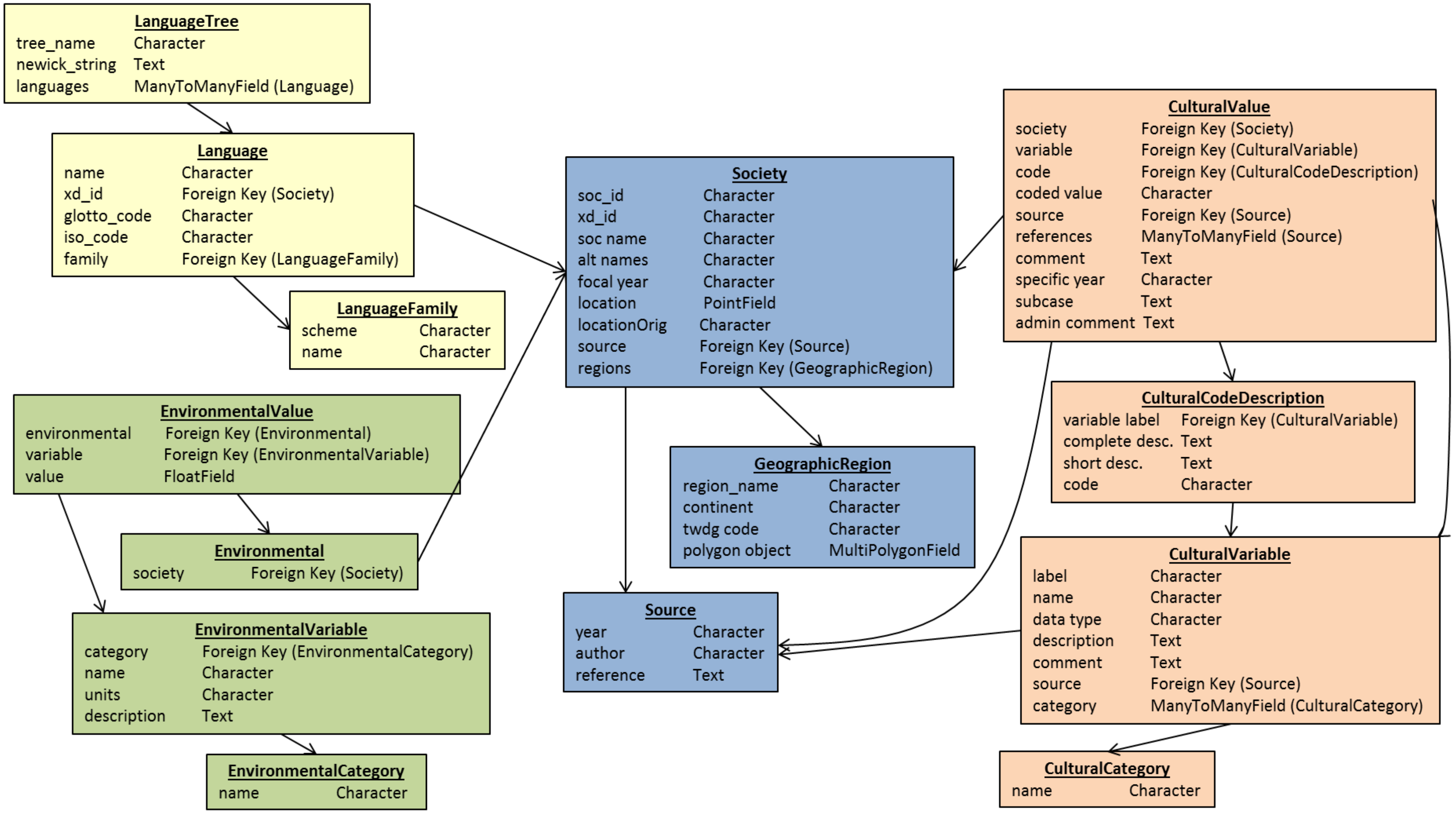

Supplement: S1 Fig — (PDF) [file pone.0158391.s001.pdf]
