# Supplementary material for: D-PLACE: A Global Database of Cultural, Linguistic and Environmental Diversity
Source: PLoS One. 2016 Jul 8;11(7):e0158391. doi: 10.1371/journal.pone.0158391 (PMC4938595; doi:10.1371/journal.pone.0158391)
Supplement: S1 Supporting information — (PDF) [file pone.0158391.s002.pdf]

## S1 Supporting information: D-PLACE data and sources

### Ethnographic Atlas data

The data were drawn from tables of coded cultural data published in twenty-nine installments in the journal *Ethnology* by George P. Murdock [1,2]. We scanned, digitized and compiled the data in these original publications, ensuring that each society was represented by the latest correction to its respective data (for a summary of publication and correction dates by society, see [3]). We then converted Murdock's letter codes and variables to those used by Gray [3]. We chose to use the Gray codes and variable numbers for two reasons. First, they make it easier to manage linked codes (Murdock's codes frequently include code "modifiers," in the form of a prefix and/or suffix (e.g., bVu, where V codes the main state, and b and u code 'alternative' states—in this case the capital letter is the main rule of marital residence, the preposited letter is the residence pattern early in the marriage and the postposited letter is a frequent alternative form). For the most part, these modifiers were transformed into stand-alone variables in Gray [3] (in this case breaking the single variable for marital residence into three, describing either the early, dominant, or alternate pattern for marital residence). Second, this process allowed us to identify errors introduced by the scanning and optical character recognition process by checking our digitized dataset against that of Gray [3]. Any mismatches were manually checked, and Murdock's entry used where a discrepancy with Gray [3] was not explained by the scanning/digitizing process. Any changes to the original data are carefully noted and explained in a "change" column associated with each variable.

Gray's [3] "Corrected Ethnographic Atlas" represented a leap forward in terms of the accessibility of the EA, and has formed the basis for many hundreds of papers in the field of

cross-cultural analysis. We have made a small number of changes to the societies, variables and codes in Gray [3], which we briefly outline here.

**Societies**: First, we deleted all data for three societies from Gray [3] that we identified as doubles of existing societies, despite their distinct identifiers: Tokelau (Ii11, double of Ii6), Chilcotin (Na18, double of Nd8) and Ojibwa (Nf1, double of Na34). We then added data for 27 societies in Eurasia to the 1264 EA societies in Gray's dataset. Data for these societies were recently coded [4,5], and fill important regional gaps in northern Eurasia.

**Cultural variables**: D-PLACE does not included variables from Gray [3] that describe geography, environment or language, as these data have been extracted for all D-PLACE societies using contemporary datasets as described in the main paper and following sections. We do, however, retain four cultural variables that were not part of the original EA but that Gray included in his Corrected EA [6]. These are Gray's Political Integration (v90) and Political succession (v94), which are available for approximately 300 societies each and were first described and published by Murdock in 1957 as part of his World Ethnographic Sample, a precursor of the EA [7]; and Trance states (v112) and Societal rigidity (v113) [8,9], for which data are available for just over 650 and 30 societies, respectively. Each variable name is also tagged with the description given to it by Murdock, which we felt often contained detail critical to new users' understanding of the data. For example, the variable described only as "Fishing" in Gray [3], is described in Murdock [1] as the dependence of a society on fishing, "including shellfishing and the pursuit of large aquatic animals".

**Codes**: While we have largely adopted the codes from Gray [3], we have further elaborated efforts in that revision to make each Murdock prefix and suffix into "stand alone" variables, and have added codes in cases where we felt missing data were not clearly differentiated from other

types of data. For example, a number of variables in Gray [3] that had been modifiers in Murdock's original EA were coded "9: Same as previous variable" in Gray [3]. We recoded these cases with the "previous" codes, instead of requiring users to refer to another variable. We also ungrouped any Murdock codes that were grouped in Gray [3], to minimize loss of information. For example, in Gray [3], "Prevailing Type of Dwelling: Wall Material (v81)" assigned code "0" to three Murdock codes: "No walls, or roof and open walls" (Murdock code R), "Walls indistinguishable from roof" (Murdock code O), and "Missing data" (Murdock code "."). We re-assigned each of the first two options its own numeric code, to preserve the detail provided by Murdock. Throughout the dataset, "missing data" was recoded as "NA" from "0". Finally, we have also returned to the longer code definitions provided by Murdock in his original publications, as these often include details omitted from the more succinct code definitions in Gray [3]. These complete code definitions are displayed in results tables, with shortened definitions' appearing in map and tree legends. All code definitions are also available in the codebooks we have made available for download on the D-PLACE website.

Two other changes to Gray [3] are worth mentioning. First, we identified errors in the focal years reported in Gray [3] for a number of societies, and therefore replaced Gray's focal year data (v102) with the focal year reported by Murdock in his original presentation of the society, or, where missing, by Ember et al. [10]. Second, Murdock (and, therefore, Gray) provided geographic coordinates for societies rounded to the nearest degree. Because the accuracy of our environmental data relies on the precision of these coordinates, we used the following approach to improve the latitude-longitude data for EA societies. First, for societies that were mapped as falling in the ocean, we manually moved the society's coordinates to the nearest piece of land, verifying the appropriate island/atoll for small-island nations using online sources and original ethnographies wherever possible. We used the Global Administrative Areas GADM v2.6 shapefile for our "land" layer (http://www.gadm.org, Last accessed November 2015).

Second, we corrected obvious errors for other, non-coastal societies (e.g., societies that were placed in the wrong hemisphere because of an inverted longitude sign). Environmental data (see below) were extracted using these revised coordinates. Interested users can identify societies that had their coordinates revised by comparing a society's "original" latitude and longitude with its revised latitude and longitude: both are included in CSV downloads of D-PLACE search results.

A final strength of the EA dataset as it is presented in D-PLACE is the linking of Murdock's coding notes with the societies and codes to which they refer. When users download the data, the comments associated with a given code are readily visible. As White [11], noted: "[Murdock's] notes provide a wealth of information useful to researchers seeking to dispute Murdock's codings or to understand his coding decisions." A complete codebook for our revised EA dataset is available for download on the D-PLACE site. We encourage users interested in the history of the EA to consult Murdock [7], in which he outlines the rationale used in selecting societies for the World Ethnographic Sample, a precursor to the EA. In particular, he makes an argument for including societies that represent "all the culture areas and subareas of the world", including "examples of recorded ancient civilizations, of the contemporary complex civilizations of Europe and Asia, of European and African cultures transplanted to other continents, and of acculturated native peoples on the same basis as indigenous ethnographic cultures" (see also the introductory sections to the Ethnographic Atlas monograph, Murdock [12]). An accessible overview of the history of the EA is available through websites maintained by Douglas White (e.g., [11]).

## Binford Hunter-Gatherer data

The data were scanned and digitized from tables of coded cultural data published in Binford [13] (tables 5.01, 6.03, 8.01, 8.04, 8.07, 8.08 and 9.01). As in the case of the EA, we retained only

those variables that describe the cultural practices of societies, leaving out ecological descriptors, as one of the goals of D-PLACE is to provide harmonized, high-quality environmental data for each society in the database. Where the same variable appeared in multiple tables, we provide the values that were listed with their source references, rather than summary values appearing in overview tables. A note on any discrepancy is included in the 'AdminComments' field that accompanies data downloads. Data for focal year, and some variable names and definitions were obtained from documentation accompanying the "ENVCALC2" programme [14]. We also exclude most variables that are easily derived from others in the dataset, or for which definitions could not be ascertained from Binford [13] or from documentation accompanying ENVCALC2 [14].

Where Binford provided multiple estimates for a single variable and society (e.g., estimates of household size at different points in time, in different seasons, or different communities; see main paper for more discussion), we have included all estimates, tagging each with its 'specific subcase' information (e.g., with the particular year, season or village to which the estimate corresponds). We encourage users interested in societies in the Binford dataset to visit the webpage of Amber L. Johnson [15], which provides additional background on the Binford Hunter-Gatherer dataset, as well as links to data for additional variables not included in Binford [13], and not currently in D-PLACE.

## Environmental data:

Geographic coordinates were obtained from the source datasets and revised where needed as described in the "Cultural data" section, above. Both the original and revised latitude and longitude are stored for all societies in the society table, and are included in CSV downloads from the site. For each society, we computed the mean, variance, and predictability of the entire

annual cycles of precipitation and temperature based on monthly global maps (0.5 by 0.5 degree cells) obtained from the CRU-TS 3.1 Climate Database [16]. Predictability was measured via Colwell's [17] Constancy, Contingency and Predictability indexes. These indexes capture the extent to which yearly cycles vary among years in terms of onset, intensity and duration, ranging from 0 (completely unpredictable) to 1 (fully predictable). We include constancy (the extent to which a variable can be predicted because it tends to stay fairly constant) and contingency (the extent to which predictions are possible because environmental cycles are highly repeatable) in order to allow interested users to explore the potentially different impacts of these two types of predictability. Monthly net primary production data were obtained from the MODIS dataset [18](data range: 2000-2016). From these data we computed the annual mean, variance predictability, constancy and contingency of net primary productivity at each sampled locality. Estimates of the number of species at each site were also obtained for birds, mammals, and amphibians from Jenkins et al. [19] and for vascular plants from Kreft and Jetz [20]. Ecoregion and biome data were obtained from Olson et al. [21] and elevation data were obtained from the GTOPO30 data set [22]. Finally, we calculated each society's distance from the coast in kilometers, using the coastline defined in the full-resolution Global Self-consistent, Hierarchical, High-resolution Geography Database [23].

**References**

1. Murdock GP. Ethnographic Atlas, Installments I-XXVII. Ethnology. 1-10.

2. Barry III H. Ethnographic Atlas XXVIII. Ethnology. 1980;19: 245–263.

3. Gray JP. A corrected ethnographic atlas. World Cult. 1999;10: 24–85.

4. Korotayev A, Kazankov A, Borinskaya S, Khaltourina D, Bondarenko D. Ethnographic atlas XXX: Peoples of Siberia. Ethnology. 2004;43: 83–92.

5. Bondarenko D, Kazankov A, Khaltourina D, Korotayev A. Ethnographic atlas XXXI: Peoples of easternmost Europe. Ethnology. 2005; 261–289.

6. Murdock GP, Textor R, Barry III H, White DR, Gray JP, Divale WT. Ethnographic Atlas Revised by World Cultures: EA01-09.SAV (digital file). World Cultures; 1999.
7. Murdock GP. World Ethnographic Sample. Am Anthropol. 1957;59: 664–687. doi:10.1525/aa.1957.59.4.02a00090
8. Bourguignon E. Religion, altered states of consciousness and social change. Columbus: Ohio State University Press; 1973.
9. Greenbaum L. Societal correlates of possession trance in Sub-Saharan Africa. In: Bourguignon E, editor. Religion, atered states of consciousness and social change. Columbus: Ohio State University Press; 1973. pp. 39–57.
10. Ember CR, Page H, Martin MM, O'Leary T. A computerized concordance of cross-cultural samples. New Haven: Human Relations Area Files; 1992.
11. White DR. Ethnographic Atlas [Internet]. Available: http://eclectic.ss.uci.edu/~drwhite/worldcul/atlas.htm
12. Murdock GP. Ethnographic Atlas XXII: A Summary. Pittsburgh: University of Pittsburgh Press; 1967.
13. Binford LR. Constructing frames of reference: an analytical method for archaeological theory building using ethnographic and environmental data sets. Univ of California Press; 2001.
14. Binford LR, Johnson AL. Documentation for Program for Calculating Environmental and Hunter-Gatherer Frames of Reference (ENVCALC2). Java version [Internet]. 2006. Available: http://www.mae.u-paris10.fr/arscan/Bases-de-donnees-ethnographique-et.html
15. Johnson AL. Data and program: Binford's environmental and hunter-gatherer frames of reference variables [Internet]. Available: http://ajohnson.sites.truman.edu/data-and-program/
16. Harris I, Jones PD, Osborn TJ, Lister DH. Updated high resolution grids of monthly climatic observations–the CRU TS3. 10 Dataset. Int J Climatol. 2014;34: 623–642.

17. Colwell RK. Predictability, constancy, and contingency of periodic phenomena. Ecology. 1974; 1148–1153.

18. NASA. Net Primary Productivity (1 month - TERRA/MODIS) [Internet]. Available: http://neo.sci.gsfc.nasa.gov/view.php?datasetId=MOD17A2_M_PSN

19. Jenkins CN, Pimm SL, Joppa LN. Global patterns of terrestrial vertebrate diversity and conservation. Proc Natl Acad Sci. 2013;110: E2602–E2610.

20. Kreft H, Jetz W. Global patterns and determinants of vascular plant diversity. Proc Natl Acad Sci. 2007;104: 5925–5930.

21. Olson DM, Dinerstein E, Wikramanayake ED, Burgess ND, Powell GVN, Underwood EC, et al. Terrestrial Ecoregions of the World: A New Map of Life on Earth A new global map of terrestrial ecoregions provides an innovative tool for conserving biodiversity. Bioscience. 2001;51: 933–938.

22. LP DAAC (Land Processes Distributed Active Archive Center) USGS/EROS. Global 30 arc-second elevation (GTOPO30): Long-term archive [Internet]. Available: https://lta.cr.usgs.gov/GTOPO30

23. Wessel P, Smith, W. H. F. Global Self-consistent, Hierarchical, High-resolution Geography Database (GSHHS) v2.3.4 [Internet]. 2015. Available: https://www.ngdc.noaa.gov/mgg/shorelines/gshhs.html
