# Supplementary material for: D-PLACE: A Global Database of Cultural, Linguistic and Environmental Diversity
Source: PLoS One. 2016 Jul 8;11(7):e0158391. doi: 10.1371/journal.pone.0158391 (PMC4938595; doi:10.1371/journal.pone.0158391)
Supplement: S1 Table — Currently, D-PLACE contains cultural data for over 1400 societies, drawn from two major cross-cultural datasets (the Ethnographic Atlas and Binford Hunter-Gatherer datasets). The societies are associated with 1202 unique languages and approximately 1315 dialects. Linguistic information for each society is available for download through D-PLACE, with all languages and dialects linked to Glottolog identifiers (glottolog.org; [43]). (PDF) [file pone.0158391.s003.pdf]

S1 Table. D-PLACE societies per language family. Currently, D-PLACE contains cultural data for over 1400 societies, drawn from two major cross-cultural datasets (the Ethnographic Atlas and Binford Hunter-Gatherer datasets). The societies are associated with 1202 unique languages and approximately 1315 dialects. Linguistic information for each society is available for download through D-PLACE, with all languages and dialects linked to Glottolog identifiers (glottolog.org).

Language family	Societies in Ethnographic Atlas dataset	Societies in Binford Hunter-Gatherer dataset	Unique languages	Unique dialects
Abkhaz-Adyge	2	0	2	2
Afro-Asiatic	104	0	72	91
Ainu	1	1	1	1
Algic	31	26	26	32
Alsea	1	1	1	1
Amerindian pidgin (Pidgin)	1	0	1	1
Araucanian	1	0	1	1
Arawakan	13	1	13	13
Athapaskan-Eyak-Tlingit	31	33	33	33
Atlantic-Congo	312	5	291	313
Austroasiatic	15	3	16	17
Austronesian	131	8	129	135
Aymara	1	0	1	1
Banaro	1	0	1	1
Barbacoan	1	0	1	1
Basque	2	0	1	2
Bookkeeping	1	0	1	1
Bororoan	2	1	2	2
Burushaski	1	0	1	1
Caddoan	5	0	4	4
Cariban	14	1	12	15
Central Sudanic	14	1	14	15
Chibchan	7	0	7	7
Chimakuan	1	1	1	1
Chimariko	1	1	1	1
Chinookan	2	2	2	2
Chitimacha	1	0	1	1
Chocoan	1	0	1	1
Chonan	2	2	2	2
Chono	0	1	1	1

Language family	Societies in Ethnographic Atlas dataset	Societies in Binford Hunter-Gatherer dataset	Unique languages	Unique dialects
Chukotko-Kamchatkan	3	0	3	3
Chumashan	1	1	1	1
Coahuilteco	1	1	1	1
Cochimi-Yuman	12	4	7	9
Coosan	1	1	1	1
Dizoid	1	0	1	1
Dogon	1	0	1	1
Dravidian	12	6	16	17
East Kutubu	1	0	1	1
Eastern Jebel	1	0	1	1
Eastern Trans-Fly	1	0	1	1
Eskimo-Aleut	17	27	9	23
Furan	1	0	1	1
Gagadu	0	1	1	1
Goilalan	1	0	1	1
Great Andamanese	1	1	1	1
Guahibo	1	1	1	1
Guaicurian	0	1	1	1
Guaicuruan	3	0	3	3
Guató	1	1	1	1
Gunwinyguan	1	3	3	3
Hadza	1	1	1	1
Haida	1	1	1	1
Heiban	5	0	5	5
Hmong-Mien	2	0	2	2
Huavean	1	0	1	1
Huitotoan	1	0	1	1
Ijoid	1	0	1	1
Indo-European	57	1	50	57
Iroquoian	3	0	3	3
Japonic	4	0	4	4
Jarawa-Onge	0	2	2	2
Jarrakan	0	1	1	1
Jicaquean	1	0	1	1
Jivaroan	1	0	1	1
Kadugli-Krongo	2	0	2	2
Kakua-Nukak	0	1	1	1
Karankawa	1	1	1	1

Language family	Societies in Ethnographic Atlas dataset	Societies in Binford Hunter-Gatherer dataset	Unique languages	Unique dialects
Karok	1	1	1	1
Kartvelian	3	0	2	3
Kawesqar	1	1	1	1
Keresan	6	0	2	5
Khoe-Kwadi	3	5	6	7
Kiowa-Tanoan	8	1	5	7
Kiwaian	1	0	1	1
Klamath-Modoc	2	2	1	1
Koiarian	2	0	2	2
Kolopom	1	0	1	1
Koman	1	0	1	1
Koreanic	1	0	1	1
Kunama	1	0	1	1
Kutenai	1	1	1	1
Kwerbic	2	0	2	2
Kxa	1	2	2	2
Laragia	0	1	1	1
Lencan	1	0	1	1
Lengua-Mascoy	1	0	1	1
Lower Sepik-Ramu	1	0	1	1
Maiduan	2	2	2	2
Mailuan	1	0	1	1
Mande	23	0	22	23
Maningrida	1	2	1	2
Mao	1	0	1	1
Marind	1	0	1	1
Matacoan	2	0	2	2
Mayan	10	0	10	10
Misumalpan	1	0	1	1
Miwok-Costanoan	2	3	3	3
Mixe-Zoque	2	0	2	2
Mongolic	7	0	7	7
Morehead-Wasur	1	0	1	1
Muskogean	4	0	4	4
Nakh-Daghestanian	1	0	1	1
Nambiquaran	1	1	1	1
Nara	1	0	1	1
Narrow Talodi	1	0	1	1

Language family	Societies in Ethnographic Atlas dataset	Societies in Binford Hunter-Gatherer dataset	Unique languages	Unique dialects
**Natchez**	1	0	1	1
**Ndu**	2	0	2	2
**Nilotic**	32	1	27	32
**Nivkh**	1	1	1	1
**North Halmahera**	1	0	1	1
**North-Eastern Tasmanian**	1	1	1	1
**Northern Daly**	0	1	1	1
**Nubian**	3	0	3	3
**Nuclear Eleman**	1	0	1	1
**Nuclear Torricelli**	1	0	1	1
**Nuclear Trans New Guinea**	11	0	11	11
**Nuclear-Macro-Je**	8	2	8	8
**Nyimang**	1	0	1	1
**Otomanguean**	6	1	6	6
**Páez**	1	0	1	1
**Palaihnihan**	2	2	2	2
**Pama-Nyungan**	8	40	39	40
**Panoan**	3	0	3	3
**Peba-Yagua**	1	0	1	1
**Pomoan**	3	4	3	3
**Pumé**	1	1	1	1
**Purari**	1	0	1	1
**Quechuan**	1	0	1	1
**Sahaptian**	4	4	4	4
**Saharan**	4	0	4	4
**Saliban**	1	0	1	1
**Salinan**	1	1	1	1
**Salishan**	21	22	18	21
**Sandawe**	1	0	1	1
**Sepik**	1	0	1	1
**Seri**	1	1	1	1
**Shastan**	1	1	1	1
**Shom Peng**	0	1	1	1
**Sino-Tibetan**	30	0	30	30
**Siouan**	12	3	10	12
**Siuslaw**	1	0	1	1
**Songhay**	2	0	2	2
**South Bougainville**	1	0	1	1

Language family	Societies in Ethnographic Atlas dataset	Societies in Binford Hunter-Gatherer dataset	Unique languages	Unique dialects
South Omotic	6	0	3	3
South-Eastern Tasmanian	0	1	1	1
Southern Daly	1	0	1	1
Surmic	3	0	3	3
Ta-Ne-Omotic	2	0	2	2
Tai-Kadai	1	1	1	1
Takelma	9	0	7	8
Tangkic	0	2	2	2
Tarascan	1	0	1	1
Tequistlatecan	1	0	1	1
Ticuna-Yuri	1	0	1	1
Timucua	1	0	1	1
Tiwi	1	1	1	1
Totonacan	1	0	1	1
Trumai	1	0	1	1
Tsimshian	1	2	2	2
Tucanoan	2	0	2	2
Tungusic	8	1	9	9
Tupian	11	4	14	14
Turkic	13	0	12	13
Tuu	1	4	4	4
Unclassifiable	0	1	1	1
Uralic	17	1	16	17
Uru-Chipaya	1	0	1	1
Uto-Aztecan	65	46	22	29
Wakashan	5	5	5	5
Warao	1	0	1	1
Washo	1	1	1	1
Wintuan	3	3	3	2
Worrorran	0	2	2	2
Yámana	1	1	1	1
Yana	1	1	1	1
Yanomam	4	1	4	4
Yele	1	0	1	1
Yeniseian	1	1	1	1
Yokutsan	3	3	2	3
Yuchi	1	0	1	1
Yukaghir	1	1	1	1

Language family	Societies in Ethnographic Atlas dataset	Societies in Binford Hunter-Gatherer dataset	Unique languages	Unique dialects
**Yuki-Wappo**	4	3	2	2
**Zamucoan**	1	0	1	1
**Zuni**	1	0	1	1
**Total**	1291	339	1202	1315
